# Ori-Finder 2, an integrated tool to predict replication origins in the archaeal genomes

**DOI:** 10.3389/fmicb.2014.00482

**Published:** 2014-09-15

**Authors:** Hao Luo, Chun-Ting Zhang, Feng Gao

**Affiliations:** ^1^Department of Physics, Tianjin UniversityTianjin, China; ^2^Key Laboratory of Systems Bioengineering (Ministry of Education), Tianjin UniversityTianjin, China; ^3^SynBio Research Platform, Collaborative Innovation Center of Chemical Science and EngineeringTianjin, China

**Keywords:** archaea, replication origins, Z-curve, origin recognition box, DNA replication

## Abstract

DNA replication is one of the most basic processes in all three domains of cellular life. With the advent of the post-genomic era, the increasing number of complete archaeal genomes has created an opportunity for exploration of the molecular mechanisms for initiating cellular DNA replication by *in vivo* experiments as well as *in silico* analysis. However, the location of replication origins (*oriC*s) in many sequenced archaeal genomes remains unknown. We present a web-based tool Ori-Finder 2 to predict *oriC*s in the archaeal genomes automatically, based on the integrated method comprising the analysis of base composition asymmetry using the Z-curve method, the distribution of origin recognition boxes identified by FIMO tool, and the occurrence of genes frequently close to *oriC*s. The web server is also able to analyze the unannotated genome sequences by integrating with gene prediction pipelines and BLAST software for gene identification and function annotation. The result of the predicted *oriC*s is displayed as an HTML table, which offers an intuitive way to browse the result in graphical and tabular form. The software presented here is accurate for the genomes with single *oriC*, but it does not necessarily find all the origins of replication for the genomes with multiple *oriC*s. Ori-Finder 2 aims to become a useful platform for the identification and analysis of *oriC*s in the archaeal genomes, which would provide insight into the replication mechanisms in archaea. The web server is freely available at http://tubic.tju.edu.cn/Ori-Finder2/.

## INTRODUCTION

DNA replication is one of the essential and conserved features among all three domains of life. In bacteria, DNA replication initiates from a single replication origin (*oriC*), which is often adjacent to the replication-related genes and distributed with the DnaA box motifs, whereas eukaryotic organisms exploit significantly more replication origins, ranging from hundreds in yeast to tens of thousands in human (Gao et al., 2012). Archaea are classified as a separate domain in the three-domain system, and share some similar features with both bacteria and eukaryotes (Woese and Fox, 1977). Similar to the bacteria, the *oriC*s in archaea are located in the intergenic regions around the replication-related proteins and distributed with the origin recognition boxes (ORBs). The ORB motifs are the conserved sequences and recognition sites for the Orc1/Cdc6 initiation proteins (Barry and Bell, 2006). In some organisms, G-stretches are also observed at the end of ORBs. On the other hand, the origin binding proteins in archaea are homologous to the related eukaryotic Orc1/Cdc6 proteins, and some archaea could also adopt more than one *oriC* to initiate DNA replication. With the increasing availability of complete archaeal genomes, identification of their *oriC*s would provide further insight into the mechanism of DNA replication in archaea and reveal the evolutionary history between bacteria and eukaryotes (Barry and Bell, 2006; Wu et al., 2014b).

The first putative *oriC* of archaea was identified in *Halobacterium* sp. strain NRC-1 by GC-skew method and demonstrated by cloning into a non-replicating plasmid (Myllykallio et al., 2000). The Z-curve method is an alternative technique that detects the asymmetrical nucleotide distribution around replication origins. The three components of the Z-curve, *x*_n_, *y*_n,_ and *z*_n_ display the distributions of purine versus pyrimidine (R vs. Y), amino versus keto (M vs. K) and strong H-bond versus weak H-bond (S vs. W) bases along the sequence, respectively. The *x*_n_ and *y*_n_ components are termed the RY and MK disparity curves, respectively. The AT and GC disparity curves are defined by (*x*_n_ + *y*_n_)/2 and (*x*_n_ -*y*_n_)/2, which shows the excess of A over T and G over C, respectively, along the sequence (Zhang and Zhang, 2005; Gao, 2014). Based on the Z-curve analysis, we have identified single *oriC* in *Methanocaldococcus jannaschii* and *Methanosarcina mazei*, double *oriC*s in *Halobacterium* sp. strain NRC-1, and three *oriC*s in *Sulfolobus solfataricus* P2, which are consistent with the subsequent experiments (Soppa, 2006). Recently, multiple *orc*1/*cdc*6-associated *oriC*s in all the available haloarchaeal genomes have been predicted by identification of putative ORBs (Wu et al., 2012). Based on these discoveries, several basic features of the *oriC*s could be summarized in archaea. Firstly, most *oriC*s are located in proximity to the genes encoding archaeal replication-related proteins, such as archaeal Orc/Cdc6 protein, Whip (Winged-Helix Initiator Protein) and DNA primase. Secondly, *oriC*s are often located around the extremes of disparity curves. Finally, most of the *oriC*s contains the AT-rich unwinding elements and conserved ORBs (Zhang and Zhang, 2005; Barry and Bell, 2006; Wu et al., 2014a).

Our group has developed a web-based system Ori-Finder 1 to find *oriC*s in the bacterial genomes based on the Z-curve method with high accuracy and reliability (Gao and Zhang, 2008). Now with the knowledge of *oriC*s in the archaeal genomes, we present an online tool, Ori-Finder 2, to identify the *oriC*s in the archaeal genomes, based on the integrated method comprising the analysis of base composition asymmetry using the Z-curve method, the distribution of ORB elements identified by FIMO tool, and the occurrence of genes frequently close to replication origins, which is available at .

## METHODS AND IMPLEMENTATION

**FIGURE 1:**
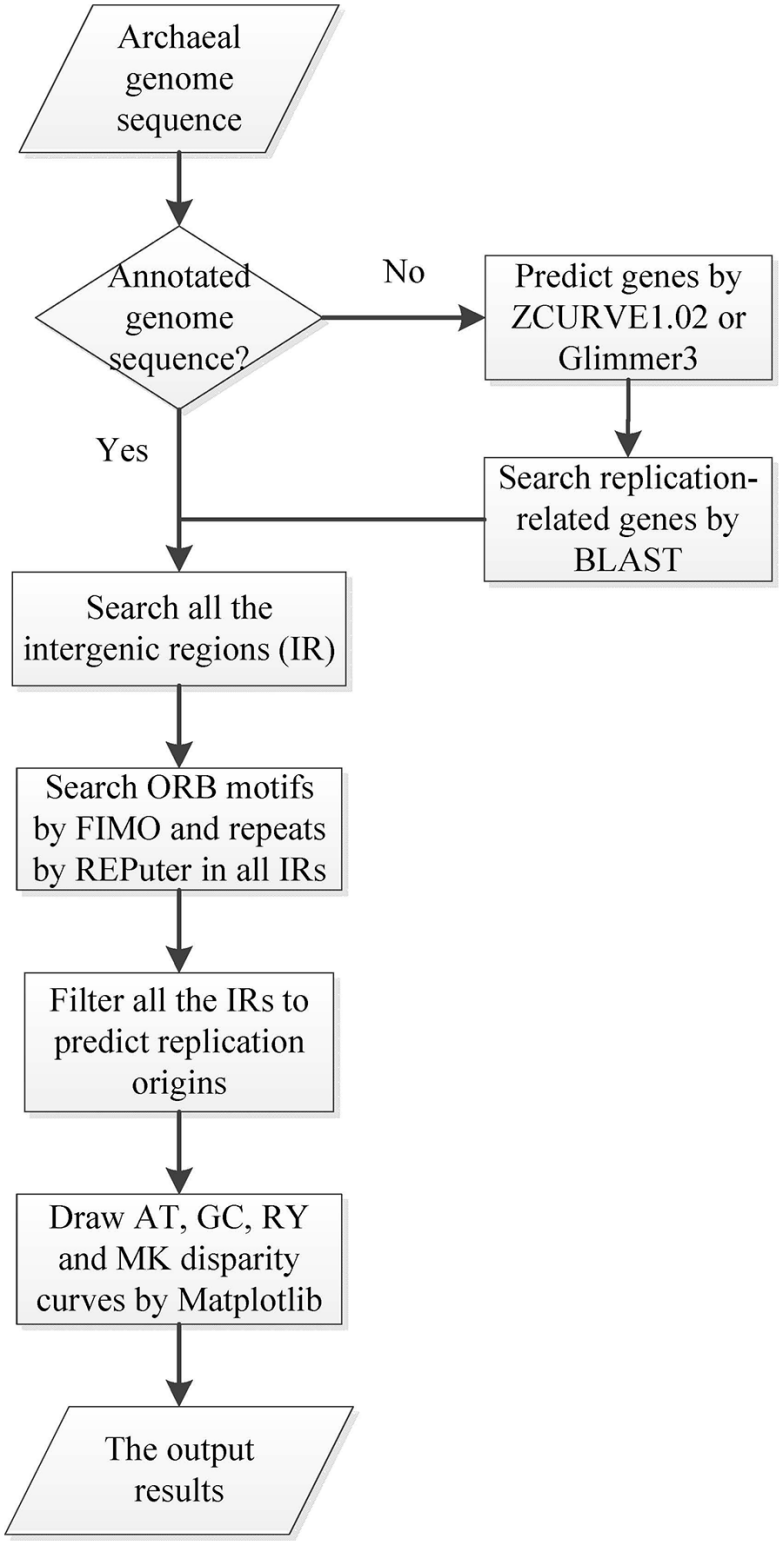
**Workflow diagram of Ori-Finder 2.** The flow chart schematically shows the procedure to identify *oriC* regions by Ori-Finder 2.

Ori-Finder 2 utilizes an integrated approach to predict *oriC*s in the user-supplied archaeal genomes automatically. **Figure 1** presents the workflow of Ori-Finder 2. Users submit an annotated or unannotated genome sequence to the web server. For the annotated genome, we recommend that users submit the sequence file in GenBank format or upload the sequence file in FASTA format as well as its corresponding protein table (PTT) file. The web server is also able to analyze the unannotated genomes by integrating two gene prediction pipelines, ZCURVE1.02 and Glimmer3 (Guo et al., 2003; Delcher et al., 2007), for gene identification and BLAST program for functional annotations of genes. Then all the intergenic sequences are scanned by Find Individual Motif Occurrences (FIMO), a software tool for scanning DNA or protein sequences with motifs described as position-specific scoring matrices (Grant et al., 2011), to obtain the ORB sequences, and also by REPuter program, a classic pipeline to compute exact repeats and palindromes in complete genomes (Kurtz et al., 2001), to identify the repeats. Finally, all the intergenic sequences adjacent to the replication-related genes with the ORB sequences are predicted as *oriC*s. Since the approach relies on the prior knowledge of *oriC*s in archaea, it may fail to identify the *oriC*s adjacent to the unknown genes which might be involved in DNA replication. In order to overcome the drawback, the intergenic sequences, which contain more than two conserved motifs, will be also predicted as *oriC*s. BLAST searches are performed against DoriC, a database of bacterial and archaeal replication origins, to search the homologs (Gao and Zhang, 2007; Gao et al., 2013). Here, the conserved motifs of ORB sequences used in FIMO were obtained from DoriC. All the records in DoriC were organized into several taxonomic clusters, including *Methanobacteriaceae*, *Methanomicrobia*, *Methanococcaceae*, *Sulfolobaceae* and *Thermococcaceae*. And the conserved ORB motifs were calculated from the corresponding clusters by Multiple EM for Motif Elicitation (MEME) program, a tool used to discover motifs in a group of related DNA or protein sequences (Bailey et al., 2009). **Table 1** displays the regular expressions of ORB motifs. Note that the common motif is calculated from all the records in DoriC. The motif logos are shown in the submission form, and the position specific probability matrix (PSPM) is available in the document webpage. Each job of Ori-Finder 2 is assigned a unique ID, and the whole process will take several minutes to complete. Users could retrieve their results with the job ID or be notified by email if specified in the submission page.

**Table 1:** The regular expressions of the ORB motifs identified by MEME.

Taxonomy	*E*-value	Regular expressions
*Halobacteriaceae*	4.5E-180	TT[TC]CACC[GCT]GAAAC[GA][AC][GA]G[GT]G[GT]
*Methanobacteriaceae*	4.20E-68	TT[TA]CACTTGAAAT[GTA]T[CT][CG]TC
*Methanomicrobia*	1.50E-202	TCCA[GC]T[GT]GAAA[CT][AG]A[AT]GGGGT
*Methanococcaceae*	4.80E-90	TT[TA][GT]ATTCA[TC][GA]AT[AT]T[AT]T[AT]
*Sulfolobacea*	7.50E-296	[GC]GGCCGG[AG]A[GT][CT][GT]T[CG]A[CA]CC[TC]GG
	2.40E-286	TCCA[AG][AT][TG]GAA[CA][CT][GA]AAGGGGT
	8.20E-120	GAGTGC[GT]CGGTT[CGT]GCA[ATC]CC[AG]
*Thermococcaceae*	9.10E-223	[TC]TCCAGTGGAAA[TC][GA]AA[AG]CTC
	6.90E-56	[CAG]TTTCCA[CT][TA]GGA[AT][CGA][CT]
	2.00E-52	AATG[ACT]ACA[AT]A[AGT]ATG[TA][TG]CATT
Common^a^	1.20E-225	TCCA[CG]T[TG]GAAA[TC][GA]AAGGGGT

a Note that the Common motif is calculated from all the records in DoriC by MEME. In *Halobacteriaceae*, *Methanobacteriaceae*, *Methanomicrobia*, *Sulfolobacea*, and *Thermococcaceae*, they share the consensus sequences “TCCA—GAAAC” similar to the common motif. In *Methanomicrobia* and *Sulfolobacea*, “G-string” (GGGGT) is observed obviously at the end of ORB motifs.

In the result, the information including genome size, GC content, the locations of replication-related genes and the predicted *oriC*s, as well as the Z-curve (AT, GC, RY, and MK disparity curves) for the input genome is displayed as an HTML table. In addition, the detailed information about the repeats identified by REPuter program, ORBs recognized by FIMO and the homologs in DoriC are also presented in the corresponding subtable. The ORB motifs in all the intergenic regions are also available for download from the provided URL. Users could also click to enlarge the embedded figure to obtain the high resolution one which displays the RY, MK, GC, AT disparity curves, replication-related proteins, and the predicted *oriC*s. The result webpage and figures will be stored in 7 days on the web server.

Ori-Finder 2 is developed using Python and PHP on a Unix platform with an Apache web-server. The web interface is implemented using Common Gateway Interface (CGI) python scripts, and the webpage is designed with HTML, CSS, and JavaScript. The pipeline of Ori-Finder 2 uses the Biopython library, and the output graphs are generated by the Python module Matplotlib (Hunter, 2007; Cock et al., 2009).

## RESULTS AND DISCUSSION

**FIGURE 2:**
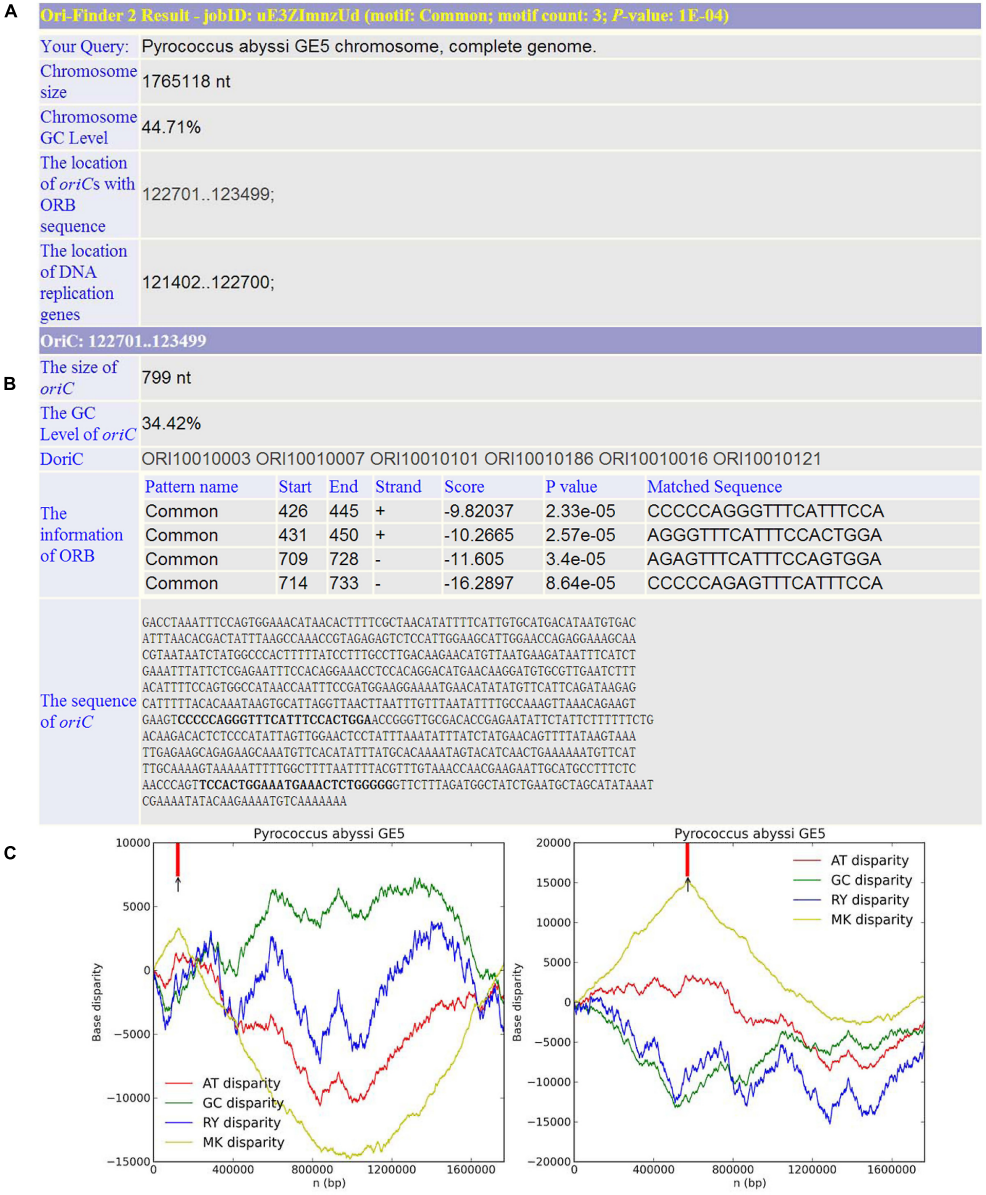
**Example of Ori-Finder 2 result for *Pyrococcus abyssi* GE5.(A)** The information of genome size, GC content, the locations of replication-related genes and the predicted *oriC*s. **(B)** The detailed information of the predicted *oriC* region including size, GC content, homologs in DoriC and sequence, as well as the information of the identified ORBs including the ORB motif (also referred to as “Pattern name”), location, strand, the associated log-likelihood ratio score, *P* value and the matched sequences. Note that the log-likelihood ratio score and *P* value are computed by FIMO to measure the similarity between the ORB motif and the matched sequence, and the *P* value cutoff for FIMO motif searching is 10^-4^. The ORB motif used here is the common motif. **(C)** The left figure shows the Z-curves (AT, GC, RY, and MK disparity curves) for the original sequence, and the right figure shows the Z-curves (AT, GC, RY, and MK disparity curves) for the rotated sequence beginning and ending in the maximum of the GC disparity curve. The short vertical red line indicates the location of replication-related protein. The black arrow is the predicted *oriC* region.

Based on this online system, we predicted the *oriC*s for all the available complete archaeal genomes in GenBank. For example, *Pyrococcus abyssi* is a classical model of DNA replication in the archaeal organisms. Similar to bacteria, there is only one *oriC* in its circular chromosome, which has been identified by cumulative oligomer skew and confirmed by *in vivo* method. With the annotated genome file, the *oriC* predicted by Ori-Finder 2 is in accordance with the experimental result and located at the peak of the MK disparity curve. Several ORB sequences are recognized in the *oriC*. **Figure 2** is a screenshot of the result by Ori-Finder 2. In addition, some archaea adopt more than one *oriC* during the DNA replication. For this situation, Ori-Finder 2 also predicted multiple *oriC*s in their genomes. *Haloferax volcanii* DS2 has a chromosome with multiple *oriC*s. Five *oriC*s were identified *in silico*, and three of them have been confirmed *in vitro* (Norais et al., 2007; Wu et al., 2012; Hawkins et al., 2013). With the annotated genome file, all the five *oriC*s mentioned above have been predicted by Ori-Finder 2 successfully, and another *oriC* with three ORB motifs is also found, which is adjacent to the genes *purO* and *cgi*. Besides that, the *oriC*s identified in the unannotated genomes are consistent with the previous results. In order to estimate the performance of Ori-Finder 2, we used 13 annotated archaeal chromosomes, whose *oriC*s have been confirmed by experimental method or identified *in silico* by other groups (**Table 2**). Compared with the records in DoriC, the sensitivity and precision are 66.7% and 62.1%, respectively. The reason of the lower precision and sensitivity compared with the programs to detect bacterial origins, such as Ori-Finder 1, is that bacteria have only one *oriC* in their chromosomes, but archaea tend to have more than one. Furthermore, *oriC*s in archaea show more diversity than those in bacteria, such as more complex ORBs in comparison with the DnaA boxes, and more unknown species-specific replication-related genes. It is difficult to predict the *oriC*s in archaea with high precision and sensitivity due to the limited amount of experimental data. For example, not all the *oriC*s in the genomes with multiple *oriC*s are found, and the ORBs with unique features need to be further explored by experimental methods. For the convenience of users’ query, the *oriC*s confirmed by *in vivo* or *in silico* methods have been collected into DoriC, which is freely available at .

**Table 2:** The prediction results of 13 archaeal chromosomes^a^.

Organism	Refseq	*OriC*s in DoriC	*OriC*s predicted by Ori-Finder 2	True positive
*Aeropyrum pernix* K1	NC_000854	2	2	1
*Pyrococcus abyssi* GE5	NC_000868	1	1	1
*Methanothermobacter thermautotrophicus* str. Delta H chromosome	NC_000916	1	2	1
*Archaeoglobus fulgidus* DSM 4304	NC_000917	1	1	0
*Pyrococcus horikoshii* OT3	NC_000961	1	1	1
*Halobacterium* sp. NRC-1	NC_002607	2	4	2
*Pyrococcus furiosus* DSM 3638	NC_003413	1	1	1
*Hyperthermus butylicus* DSM 5456	NC_008818	2	1	1
*Pyrobaculum calidifontis* JCM 11548	NC_009073	1	1	0
*Haloferax volcanii* DS2	NC_013967	5	6	5
*Haloarcula hispanica* ATCC 33960 chromosome II	NC_015943	4	7	3
*Haloarcula hispanica* ATCC 33960 chromosome I	NC_015948	5	1	1
*Nitrosopumilus maritimus* SCM1	NC_010085	1	1	1
Total	–	27	29	18

a Note that the detailed information is available at http://tubic.tju.edu.cn/Ori-Finder2/doc.php#9.

## CONCLUSION

Here, we presented a user-friendly interactive web-based platform Ori-Finder 2 to predict the *oriC*s in the archaeal genomes. The tool integrated several genomic pipelines, including FIMO, BLAST, ZCURVE, Glimmer, and REPuter, to comprehensively annotate and analyze the *oriC*s. Moreover, the ORB motifs are also calculated by MEME and organized by taxonomy. The software presented here does not necessarily find all the origins of replication in cases where there are multiple ones in a genome. However, we will continually strive to improve our approach to make it more accurate and sensitive with the increase of the *oriC*s confirmed experimentally in archaea. As the only currently available auto-annotation system for the archaeal replication origins at the sequence level, we believe that Ori-Finder 2 will be helpful to predict the archaeal replication origins and provide insight into DNA replication in archaea.

## AUTHOR CONTRIBUTIONS

Hao Luo designed the computer program and drafted the manuscript. Chun-Ting Zhang and Feng Gao supervised the study and revised the manuscript. All authors read and approved the final manuscript.

## Conflict of Interest Statement

The authors declare that the research was conducted in the absence of any commercial or financial relationships that could be construed as a potential conflict of interest.
